# Biological functions of 12(*S*)-hydroxyheptadecatrienoic acid as a ligand of leukotriene B_4_ receptor 2

**DOI:** 10.1186/s41232-018-0087-4

**Published:** 2018-10-29

**Authors:** Toshiaki Okuno, Takehiko Yokomizo

**Affiliations:** Department of Biochemistry, Juntendo University School of Medicine, Tokyo, Japan

**Keywords:** 12-HHT, Barrier function, BLT2, Cyclooxygenase, GPCR, Leukotriene B_4_, NSAID, Prostaglandin

## Abstract

Although 12(*S*)-hydroxyheptadecatrienoic acid (12-HHT) is an abundant fatty acid, it is long considered a byproduct of thromboxane A_2_ production. We identified a leukotriene B_4_ receptor 2 (BLT2)-specific agonistic activity in lipid extracts from rat small intestine, and mass spectrometric analysis of partially purified lipids containing BLT2 agonistic activity revealed that 12-HHT is an endogenous ligand of BLT2. In a dextran sulfate sodium (DSS)-induced inflammatory colitis model, BLT2-deficient mice exhibited enhanced intestinal inflammation, possibly due to impaired epithelial barrier function. In a skin wound healing model, BLT2-deficient mice exhibited delayed wound healing via dampened keratinocyte migration. BLT2 also accelerates corneal wound healing, and eye drops containing a non-steroidal anti-inflammatory drug (NSAID) inhibit the production of 12-HHT, resulting in delayed corneal wound healing. Furthermore, BLT2 is expressed in pulmonary epithelial type II cells and vascular endothelial cells in the mouse lung, and BLT2-deficient mice are more susceptible to lung damage by pneumolysin. In this review, we summarize the identification and characterization of 12-HHT as a ligand for BLT2 and discuss recent research on the physiological and pathophysiological roles of the 12-HHT-BLT2 axis. Some side effects of NSAIDs such as delayed wound healing may be caused by reduced 12-HHT production rather than diminished production of prostaglandins.

## Background

**Fig. 1:**
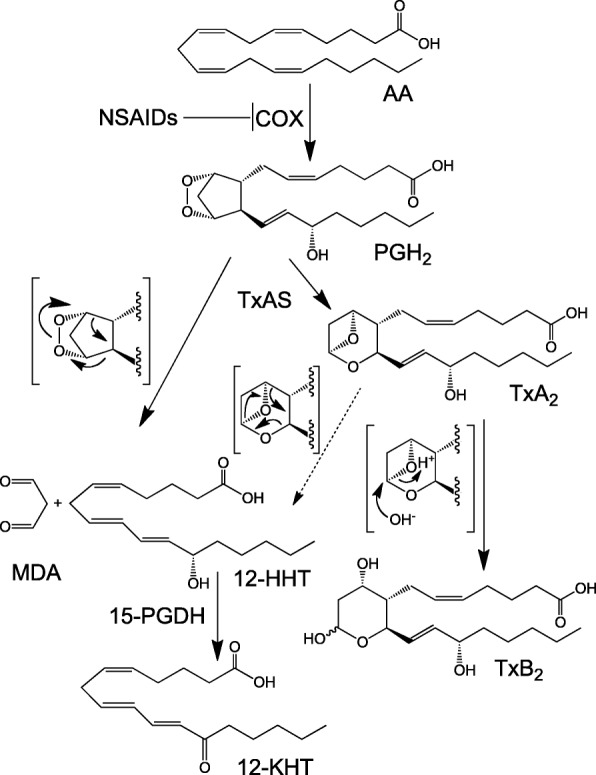
Biosynthesis and metabolic pathways of 12(*S*)-hydroxyheptadecatrienoic acid (12-HHT). Thromboxane (Tx) A_2_ synthase (TxAS) catalyzes the fragmentation of PGH_2_ into 12-HHT and malondialdehyde (MDA). TxA_2_ is unstable in aqueous solution and rapidly hydrolyzed to TxB_2_, but a proportion of TxA_2_ is hydrolyzed to 12-HHT and MDA. 12-HHT is metabolized to 12-keto-heptadecatrienoic acid (12-KHT) by 15-hydroxyprostaglandin dehydrogenase (15-PGDH)

The prostaglandin (PG) H_2_ metabolite 12(*S*)-hydroxyheptadecatrienoic acid (12-HHT, Fig. 1) is biosynthesized by cyclooxygenase (COX) from arachidonic acid [1]. Some G protein-coupled receptors (GPCRs) related to PGs and leukotrienes (LTs), and metabolites of arachidonic acid (AA), were identified in the 1990s [2, 3]. By generating and analyzing gene-deficient mice in which receptors and biosynthetic enzymes for PGs and LTs were disrupted, the biological significance of PGs and LTs has been elucidated [4]. 12-HHT was identified in the 1960s, but it was considered merely as a byproduct of thromboxane (Tx) A_2_ production [5]. In 2008, we revealed that 12-HHT is an endogenous ligand of BLT2, originally identified as a low-affinity GPCR for leukotriene B_4_ (LTB_4_) [6]. Our recent studies demonstrated that the 12-HHT-BLT2 axis contributes to the epithelial barrier functions of small intestine [7], skin [8], lung [9], and cornea [10]. In this review, we summarize the identification of 12-HHT as a ligand for BLT2, together with recent knowledge of the biological functions of the 12-HHT-BLT2 axis.

### Identification of 12-HHT as a natural ligand of BLT2

The second LTB_4_ receptor, BLT2, was first identified as a low-affinity receptor for LTB_4_ [11]. Due to the high concentration of LTB_4_ required for BLT2 activation, we hypothesized that BLT2 might have a high-affinity lipid ligand besides LTB_4_. To identify the bona fide ligand of BLT2, we extracted lipids from several rat organs and examined their agonistic activities using Chinese hamster ovary (CHO) cells expressing human BLT2. The acetone-soluble fraction of lipids extracted from rat small intestine exhibited a strong agonistic activity toward BLT2. Lipids were separated by high-performance liquid chromatography (HPLC) and the agonistic activities of fractions toward BLT2-expressing CHO cells were analyzed with a cytosensor microphysiometer that detects acidification of the extracellular fluid caused by proton efflux from activated cells. The fraction containing a strong agonistic activity was analyzed by mass spectrometry (MS) to elucidate the molecular mass and structure of the BLT2 agonistic lipid therein. The combination of exact mass measurement and MS/MS analysis revealed the BLT2 agonist as 12(*S*)-hydroxyheptadecatrienoic acid (12-HHT), a C17 fatty acid. Commercially available 12-HHT from both Cayman and Biomol (Enzo) activated CHO-BLT2 at a lower concentration than LTB_4_ in calcium, cAMP, and chemotaxis assays. The HPLC retention time of authentic 12-HHT and *m*/*z* values of MS/MS fragments were identical to those of the agonist extracted from rat small intestine. Furthermore, lipids extracted from small intestine of COX-1-deficient mice displayed much lower agonistic activity than wild-type (WT) mice, suggesting that 12-HHT, a metabolite of COX-1, is an endogenous ligand of BLT2 [6]. Recently, we cloned two zebrafish orthologues of human BLT2, zBLT2a, and zBLT2b, and these receptors were also activated by a lower concentration of 12-HHT than LTB_4_, in a similar fashion to human and mouse BLT2 [12].

### Biosynthesis and metabolism of 12-HHT

12-HHT is biosynthesized from the AA metabolite PGH_2_ by COX. Thromboxane A synthase (TxAS) catalyzes not only the rearrangement of PGH_2_ to TxA_2_, it also catalyzes in parallel, and to an almost equimolar amount, its fragmentation into 12-HHT and malondialdehyde (MDA) [13, 14]. During platelet aggregation, large amounts of TxA_2_ and 12-HHT are produced by the actions of cytosolic phospholipase A_2_α(cPLA_2_α), COX-1, and TxAS. 12(*S*)-Hydroxyeicosatetraenoic acid (12-HETE) is also produced from activated platelets by the action of 12(*S*)-lipoxygenase (12-LO) [15]. Non-steroidal anti-inflammatory drug (NSAID) treatment induces the shunting of AA from PG metabolism to 12-HETE production [16]. A high concentration of 12-HETE also activates BLT2 (16), but the biological significance of the 12-HETE-BLT2 axis remains elusive. In addition to catalysis by TxAS, 12-HHT is synthesized from PGH_2_ via a non-enzymatic pathway [17]. PGH_2_ is extremely unstable and rapidly hydrolyzed to 12-HHT and MDA, or PGE_2_, PGD_2_, and PGF_2_α in aqueous solution. PGH_2_ is also rapidly converted to 12-HHT and MDA in the presence of heme or glutathione [13]. TxA_2_ is also an unstable metabolite of PGH_2_, and most TxA_2_ is hydrolyzed to TxB_2_, but a proportion of TxA_2_ may be hydrolyzed to 12-HHT and MDA (Fig. 1). Additionally, the cytochrome P450 enzyme CYP2S1 that is expressed in macrophages reportedly generates 12-HHT [18, 19], but the contribution of CYP2S1 to 12-HHT production is uncertain. Hecker et al. reported that 12-HHT is preferentially metabolized to a 12-keto derivative by 15-hydroxyprostaglandin dehydrogenase (15-PGDH) [20]. We examined the agonistic activity of 12-keto-heptadecatrienoic acid (12-KHT), which was chemically synthesized by our collaborator [21], toward BLT2, and this was lower than that of 12-HHT (Okuno, unpublished). Eicosanoid profiling using LC-MS/MS with synthetic 12-KHT as a standard for multiple reaction monitoring (MRM) revealed the presence of 12-KHT in various cells and tissues in which 12-HHT is abundant, suggesting that 12-KHT is a metabolite of 12-HHT.

### Physiological and pathophysiological roles of the 12-HHT-BLT2 axis

BLT2 is expressed in epithelial cells of intestine and skin keratinocytes in mice [22], suggesting that the 12-HHT-BLT2 axis may contribute to epithelial functions (Table 1). To investigate the roles of BLT2 in intestinal epithelial cells, we analyzed a dextran sulfate sodium (DSS)-induced colitis mouse model. BLT2-deficient mice exhibited enhanced intestinal inflammation, possibly caused by impaired barrier function [7]. Madin-Darby canine kidney II (MDCK II) cells overexpressing BLT2 exhibited enhanced barrier function when measuring transepithelial electrical resistance (TER) and FITC-dextran leakage. Interestingly, BLT2 was localized to the lateral membrane, and it increased claudin-4 (CLDN4) expression via the Gαi protein-p38 MAPK pathway [23].

**Table 1:** Physiological and pathophysiological roles of the 12-HHT-BLT2 axis

Tissues/cells	In vivo roles of 12-HHT/BLT2	Possible mechanisms
Skin	Acceleration of wound healing	Keratinocyte migration
Cornea	Acceleration of wound healing	Corneal epithelial cell migration
Lung	Protective roles in acute lung injury	Inhibition of CysLT1 signaling
Lung	Suppression of asthma	Reduction of IL-13
Cancer	Chemotherapy resistance	Survival of cancer cells

**Fig. 2:**
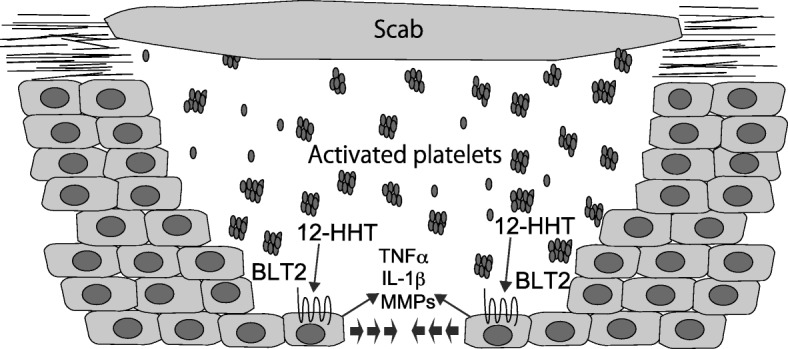
Roles of the 12-HHT-LTB_4_ receptor 2 (BLT2) axis in skin wound healing. BLT2 expressed on the surface of keratinocytes is activated by 12-HHT produced by activated platelets. The 12-HHT-BLT2 axis accelerates keratinocyte migration via the production of tumor necrosis factor (TNF)α, interleukin (IL)-1β, and matrix metalloproteinases (MMPs)

To investigate the roles of BLT2 in skin, we evaluated a skin wound healing model. BLT2-deficient mice exhibited delayed wound healing compared with WT mice. Aspirin-treated mice also displayed delayed wound healing, and the delay was abolished in BLT2-deficient mice. TxAS-deficient mice also exhibited partially delayed wound healing, but TxA_2_/PGH_2_ receptor (TP)-deficient mice did not show this phenotype. Importantly, a synthetic BLT2 agonist accelerated wound healing in C57BL/6J and diabetic *db/db* mice [8]. We also examined the detailed mechanism of BLT2-dependent acceleration of wound healing. BLT2 stimulation leads to the expression of tumor necrosis factor (TNF)α and interleukin (IL)-1β, both of which stimulate the expression and secretion of metalloproteinases (MMPs) that in turn accelerate keratinocyte migration, possibly by degrading extracellular matrix. These results suggest that the 12-HHT-BLT2 axis accelerates skin wound healing in vivo (Fig. 2). As described above, NSAIDs such as aspirin inhibit the production of 12-HHT. Our study clearly showed that aspirin-dependent delay in skin wound healing is due to the reduced production of 12-HHT, but not PGs. Reduced 12-HHT levels may therefore explain some side effects of NSAIDs.

Recently, we showed that BLT2 is also expressed in corneal epithelial cells in mice and humans, and the 12-HHT-BLT2 axis accelerates corneal epithelial cell migration and healing of corneal wounds. NSAID-containing eye drops inhibit the production of 12-HHT, which also delays corneal wound healing. These results suggest that the 12-HHT-BLT2 axis accelerates corneal wound healing in a similar manner to skin [10].

**Fig. 3:**
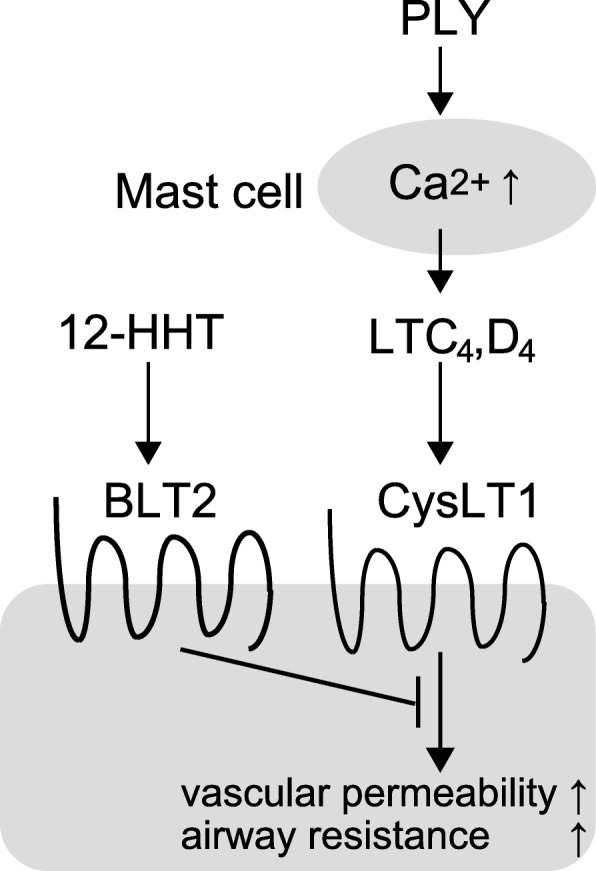
Roles of the 12-HHT-LTB_4_ receptor 2 (BLT2) axis in lung injury. PLY treatment induces the production of large amounts of cysteinyl leukotrienes (CysLTs) from mast cells. The CysLT1 receptor antagonist ameliorates PLY-induced mortality, vascular permeability, and airway resistance. 12-HHT-BLT2 axis suppresses CysLT1 signaling in vascular endothelial cells and smooth muscle cells, but the detailed molecular mechanism is under investigation

Moreover, we found that BLT2 is expressed in pulmonary epithelial type II cells and vascular endothelial cells in the mouse lung. To investigate the roles of BLT2 in lungs, mice were intratracheally treated with pneumolysin (PLY) that induces acute lung injury (ALI). Surprisingly, BLT2-deficient mice were more susceptible to lung damage by PLY, and most BLT2-deficient mice died within minutes, in contrast to intact WT mice. Although the detailed roles of BLT2 in protection against ALI are unclear, we found that PLY treatment induced the production of large amounts of cysteinyl leukotrienes (CysLTs), and a CysLT1 receptor antagonist recovered PLY-induced mortality, vascular permeability, and airway resistance, in both WT and BLT2-deficient mice. These results suggest that the 12-HHT-BLT2 axis suppresses CysLT1 signaling in vascular endothelial cells because production of CysLTs was not affected by BLT2 deficiency (Fig. 3) [9]. In addition, BLT2-knockout (KO) mice exhibited severe eosinophilic lung inflammation in an ovalbumin (OVA)-induced allergic airway disease model. This was explained by enhanced IL-13 production from BLT2-deficient CD4^+^ cells [24].

Furthermore, 12-HHT-BLT2 signaling is reported to be involved in chemotherapy resistance. F4/80^+^/CD11b^low^ splenocytes produce 12-HHT following treatment with platinum analogs, which mediates chemotherapy resistance. Interestingly, genetic loss or chemical inhibition of BLT2 prevents 12-HHT-mediated resistance [25]. Combined indomethacin and platinum-based chemotherapy may therefore improve chemo-sensitivity by reducing the production of 12-HHT [26]. We and others reported the roles of BLT2 in cancer cells. Human pancreatic cancer cells express BLT2, and treatment with the BLT2 antagonist or BLT2 knockdown inhibited proliferation and induces apoptosis in pancreatic cancer cells [27–29]. Generation of LTB_4_-BLT2-dependent reactive oxygen species (ROS) promotes anti-apoptotic, invasive, and metastatic phenotypes in cancer cells [30–33], suggesting that BLT2 antagonists might be candidates for therapeutic agents against cancer.

## Conclusions

For a long time, 12-HHT was considered merely a byproduct of thromboxane biosynthesis, and a biomarker of COX activation. However, we discovered that 12-HHT is an endogenous ligand of BLT2 using unbiased ligand screening and, with others, revealed that the 12-HHT-BLT2 axis mediates various biological functions including the epithelial barrier, wound healing, immunosuppression, and lung protection in vivo. Some side effects of NSAIDs, such as delayed wound healing, may be caused by reduced 12-HHT production rather than diminished PG production.

## Abbreviations

12-HHT: 12(*S*)-Hydroxyheptadecatrienoic acid
ALI: Acute lung injury
BLT2: LTB_4_ receptor 2
COX: Cyclooxygenase
CysLT: Cysteinyl leukotriene
LT: Leukotriene
MDA: Malondialdehyde
NSAID: Non-steroidal anti-inflammatory drug
PG: Prostaglandin
PLY: Pneumolysin
Tx: Thromboxane
